# Effects from a 90-day inhalation toxicity study with cerium oxide and barium sulfate nanoparticles in rats

**DOI:** 10.1186/s12989-017-0204-6

**Published:** 2017-07-12

**Authors:** Daniela Schwotzer, Heinrich Ernst, Dirk Schaudien, Heiko Kock, Gerhard Pohlmann, Clemens Dasenbrock, Otto Creutzenberg

**Affiliations:** Fraunhofer Institute for Toxicology and Experimental Medicine ITEM, Nikolai-Fuchs-Straße 1, 30625 Hannover, Germany

**Keywords:** In vivo, Subchronic inhalation, Nanoparticles, Cerium oxide, Barium sulfate, Inflammation, Overload, Long-term effects, Persistency

## Abstract

**Background:**

Nanomaterials like cerium oxide and barium sulfate are frequently processed in industrial and consumer products and exposure of humans and other organisms is likely. Generally less information is given on health effects and toxicity, especially regarding long-term exposure to low nanoparticle doses. Since inhalation is still the major route of uptake the present study focused on pulmonary effects of CeO_2_NM-212 (0.1, 0.3, 1.0, 3.0 mg/m^3^) and BaSO_4_NM-220 nanoparticles (50.0 mg/m^3^) in a 90-day exposure setup. To define particle-related effects and potential mechanisms of action, observations in histopathology, bronchoalveolar lavage and immunohistochemistry were linked to pulmonary deposition and clearance rates. This further allows evaluation of potential overload related effects.

**Results:**

Lung burden values increased with increasing nanoparticle dose levels and ongoing exposure. At higher doses, cerium clearance was impaired, suggesting lung overload. Barium elimination was extremely rapid and without any signs of overload. Bronchoalveolar lavage fluid analysis and histopathology revealed lung tissue inflammation with increasing severity and post-exposure persistency for CeO_2_. Also, marker levels for genotoxicity and cell proliferation were significantly increased. BaSO_4_ showed less inflammation or persistency of effects and particularly affected the nasal cavity.

**Conclusion:**

CeO_2_ nanoparticles penetrate the alveolar space and affect the respiratory tract after inhalation mainly in terms of inflammation. Effects at low dose levels and post-exposure persistency suggest potential long-term effects and a notable relevance for human health. The generated data might be useful to improve nanoparticle risk assessment and threshold value generation. Mechanistic investigations at conditions of non-overload and absent inflammation should be further investigated in future studies.

## Background

The use of nanomaterials in industry and consumer products is still intensively expanding. Due to a greater surface area per mass compared to their micro-sized counterparts, nanoparticles provide beneficial characteristics for efficient product improvement. Nanomaterials are used in many different application fields including the chemical sector, food industry as well as cosmetics and pharmaceuticals. Subjects of this study were cerium oxide and barium sulfate nanoparticles. To generate data on the safe use of such materials for manufacturers and consumers the current project was initiated and funded by the German Federal Ministry of Education and Research (03X0149). In addition, the study expands the data output of the parallel combined chronic inhalation toxicity and carcinogenicity study with CeO_2_ and BaSO_4_ in the NANoREG program (81|0661/10|170) (BASF, Ludwigshafen, Germany), especially with sensitive early mechanistic endpoints (immunohistochemistry and gene expression analysis).

Characteristics like catalytic activity give rise to the use of nano-CeO_2_ e.g. as an additional oxygen source in diesel fuel, to reduce fuel consumption and particulate emissions [1, 2]. The key benefit in this case is the higher surface area per mass ratio of the nano-sized material [3]. CeO_2_ nanoparticles are further used as a polishing agent [4]. Also, anti-oxidative effects due to ion formation on the nanoparticle surface and the resulting opportunity of its use in biomedicine is discussed [5]. However, effects of CeO_2_ nanoparticle exposure seem to be controversial since contrastingly pro-oxidative and inflammatory reactions are described. Barium sulfate is generally considered as chemically inert and non-toxic. In addition, it provides a variety of beneficial characteristics like high density and low solubility, of which e.g. plastic and paint industries take advantage. The substances CeO_2_ NM-212 and BaSO_4_ NM-220, used in this project are two well characterized nanomaterials from the European Commission Joint Research Center (JRC) nanomaterials (NM) repository (Ispra, Italy). CeO_2_ NM-212 is water insoluble with a primary particle size of 33 nm and a specific surface area of 28 m^2^/g. BaSO_4_ NM-220 also displays extremely low water solubility (0.6 × 10^−3^ w-% Ba^++^). Its primary particle size and specific surface area is 37.5 nm and 41.4 m^2^/g respectively. Both substances were tested in a short-term inhalation setup, together with 11 other nanomaterials [6]. Based on the results CeO_2_ NM-212 and BaSO_4_ NM-220 were chosen as representative nanomaterials with respectively higher and lower toxicity for further investigation regarding long-term exposure. The frequent use of nanomaterials combined with high reactivity requires appropriate assessment of potential health risks and environmental effects. Human exposure to nanoparticles during product manufacturing and application is likely. However, there is still a lack of data especially regarding long-term exposure and chronic effects of nanomaterials.

Once inhaled, particles deposit in the respiratory tract. The site of deposition depends on the material’s physico-chemical characteristics with the particle diameter as one important factor [7]. A smaller size results in penetration of deeper lung compartments. In the different areas of the respiratory tract different mechanisms of deposition are predominating. Nano-sized particles (< 100 nm) deposit in the whole respiratory tract, ending up in the alveolar region where its deposition is dominated by processes of diffusion [7–9]. However, at higher mass median aerodynamic diameter (MMAD) levels (e.g. in aerosol experiments with occupational settings, approx. 0.7 μm) sedimentation of particles plays an important role. Lung clearance of particulate matter depends on the site of deposition as well as material characteristics including solubility and bioreactivity [9, 10]. In the alveolar space the most relevant route of particle clearance is phagocytosis by alveolar macrophages and subsequent elimination primarily via the mucociliary escalator or secondarily via the lymphatic system [9, 10]. For CeO_2_ and BaSO_4_ as poorly water soluble substances, uptake and elimination by alveolar macrophages is expected to be the major clearance route. It is known that respective mechanisms could be impaired by high levels of particulate matter in the respiratory tract, when particle deposition exceeds its clearance (overload situation) [11, 12]. Persistent particle concentrations above the overload threshold eventually lead to increasing lung burden further resulting in chronic inflammation and high risks of related adverse effects like fibrosis and tumor development [11]. Particles usually have retention half-times of about 70 days [11, 13–15]. Respective periods are prolonged during lung overload [11, 14]. Based on a volumetric perspective an overload threshold of 1–2 μl PM/lung is assumed for particles with a density of 1 g/cm^3^ [12]. For CeO_2_ NM-212 an alveolar deposition fraction of about 6% has been described after a single 6 h nose-only exposure [16] and 28-day inhalation of 0.5 mg/m^3^ CeO_2_ NM-212 resulted in a retention half-time of 40 days [17].

Yokel et al. [10] in 2014 comprehensively reviewed published data on the toxicity of nano-CeO_2_ with respect to different uptake routes and exposure durations, tissue distribution and potential mechanisms of action. They confirmed the still existing lack of data regarding subchronic and chronic inhalation and clearly pointed out the inherent risk of adverse health effects due to long-term low dose CeO_2_ nanoparticle exposure. Two 90-day inhalation toxicity studies with CeO_2_ exist [18, 19]. One was performed with micro-scaled ceria indicating dose-related effects, including hyperplasia of lung tissue and related lymph nodes [19]. The only investigations on subchronic effects of nano-scaled ceria are part of a combined chronic toxicity and carcinogenicity study according to OECD TG 453 (BASF, Ludwigshafen, Germany) initiated in 2013. Recently published data from this study on genotoxicity in blood cells of exposed rats indicated absence of respective effects after 3 and 6 month periods of exposure to concentrations up to 3 mg/m^3^ CeO_2_ and 50 mg/m^3^ BaSO_4_ [18]. Further results of this study are currently pending. For barium sulfate one additional subchronic test was published, in which slight pulmonary responses after inhalation were detected [20]. Systemic distribution did not give rise to adverse effects [20]. In general it was found that despite their low solubility, after inhalation BaSO_4_ nanoparticles are cleared from the respiratory tract more rapidly compared to other poorly soluble nanoparticles, including CeO_2_ [20, 21]. The low toxic potential of BaSO_4_ is further emphasized by a 5 day inhalation study in which a no-observed adverse effect concentration (NOAEC) of at least 50 mg/m^3^ has been determined [6].

A small number of subacute inhalation studies were published, in which local effects on respiratory organs and systemic distribution of cerium oxide nanoparticles were examined [3, 16, 17, 22, 23]. Inhalation of CeO_2_induced pulmonary inflammation in a concentration-dependent manner with post-exposure persistency [3, 17, 22, 23]. Respective studies further indicated distribution of cerium to extra-pulmonary organs [16, 17, 22] and impaired nanoparticle clearance at high dose levels (≥ 5 mg/m^3^) [16, 17]. The no-observed adverse effect level (NOAEL) for CeO_2_ is expected to be below 0.5 mg/m^3^ [17]. Several short-term inhalation studies (≤ 5 days exposure) [6, 24–26] as well as examinations after intratracheal instillation [27–30] support findings like the induction of inflammatory reactions due to CeO_2_ nanoparticle exposure. Also, only a small number of the described inhalation studies covered investigations on low concentrations (< 3 mg/m^3^) of the nanomaterial [17, 18, 23]. It is unclear if low, more realistic doses of CeO_2_ nanoparticles cause similar adverse effects as exposure to high concentrations, including those exceeding the overload threshold.

The present study aimed on generating currently missing data on subchronic inhalation of CeO_2_ nanoparticles at low to moderate exposure levels and with respect to setting no effect levels. Nanoparticle concentrations should cover the induction of inflammation in the absence and presence of lung overload [17, 18]. Since it is generally estimated that barium sulfate does not cause adverse and irreversible health effects after inhalation, it was tested if this classification is even applicable for repeated exposure to a very high concentration of nano-BaSO_4_, a level at which lung overload is expected. The carcinogenicity study (BASF, Ludwigshafen, Germany) mentioned earlier serves as an important reference regarding our subchronic investigations as both studies were performed with the same substances and concentrations under similar experimental conditions. This allows correlation of early detected findings to chronic particle-related effects and might serve as basis for the identification of early biomarkers for long-term exposure health risks. Valid markers for prediction of effects in turn can help to reduce long-term in vivo studies according to the “3R Principle” for replacement, reduction and refinement of animal experiments [31].

## Results

### Aerosol characteristics

Dry powder aerosolization of nanoparticles revealed constant aerosol concentrations during 90 days exposure. Mean values were close to the required nanoparticle concentrations. Mass median aerodynamic diameters (MMAD) were determined to ensure appropriate nanoparticle size distribution. Mean values for CeO_2_ range from 0.63 to 0.79 μm. All results are listed in Table 1.

**Table 1:** Nanoparticle concentrations and MMAD values during 90-day exposure

	CeO_2_ NM-212				BaSO_4_ NM-220
NP concentration, required (mg/m^3^)	0.1	0.3	1.0	3.0	50.0
NP concentration, measured (mg/m^3^ ± SD)^a^	0.12 ± 0.04	0. 33 ± 0.09	1.06 ± 0.16	3.04 ± 0.30	48.82 ± 4.52
MMAD (μm ± GSD)^b^	0.71 ± 3.59	0.63 ± 3.83	0.68 ± 4.23	0.79 ± 3.50	2.95 ± 2.43

^a^ *n* = 78 exposure days; ^b^ *n* = 3

### (post-)exposure period and animal health

**Fig. 1:**
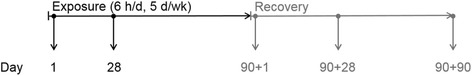
Timeline of test item exposure and sacrifices. Animals were exposed over a time period of 90 days, followed by a post-exposure period of an additional 90 days. Clinical examinations were performed after one and 28 days exposure and after one, 28 and 90 days post-exposure

Exposure of animals to the test items was performed as scheduled for 90 days with investigations on satellite groups after one and 28 days and a post exposure period of an additional 28 or 90 days (Fig. 1). All animals were in good physical conditions up to sacrifice. No significant changes in body weights or food and water consumption were detected (data not shown). Clinical signs due to particle exposure were not observed either.

### Lung burden

Based on the aerosol characteristics measured during nanoparticle exposure, a prediction of the deposited alveolar fraction was generated using the “multiple path particle dosimetry (MPPD) model” version 2.11 [32]. Based on MMAD and GSD a deposition fraction of about 10% was calculated for CeO_2_ exposure, (Table 2). For BaSO_4_ the calculated deposition fraction was 3.2%. The expected lung burden was determined with the following equations:

a $$ Dep(1)= MV\  x\ {t}_1\  x\  C\  x\  DF $$

b $$ Dep(t)=\frac{\frac{5}{7}\  Dep(1)}{k}\  x\ \left(1-{e}^{- kt}\right) $$

**Table 2:** Predicted lung burden and deposition fraction of exposed animals

	Predicted lung burden (μg/lung)			Deposition fraction (%)
	d1^a^	d28^a^	d90^a^	
0.1 mg/m^3^ CeO_2_	0.8	9.8	25.9	10.5
0.3 mg/m^3^ CeO_2_	2.5	32.2	85.0	11.5
1.0 mg/m^3^ CeO_2_	7.8	101.7	268.7	10.9
3.0 mg/m^3^ CeO_2_	20.7	286.2	862.0	9.6
50.0 mg/m^3^ BaSO_4_	115.2	1590.0	4788.9	3.2

^a^d1, d28 and d90 account for one, 20 and 65 days exposure respectively

Whereas Dep(1) = deposited mass (μg) after exposure day 1, D(t) = deposited mass (μg) after t exposure days, t = exposure time (days), t_1_ = exposure time, day 1 (min), MV = minute volume rat (l/min), C = initial nanoparticle concentration (mg/m^3^), DF = deposition fraction and k = elimination constant (k = ln(2)/t_1/2_).

**Fig. 2:**
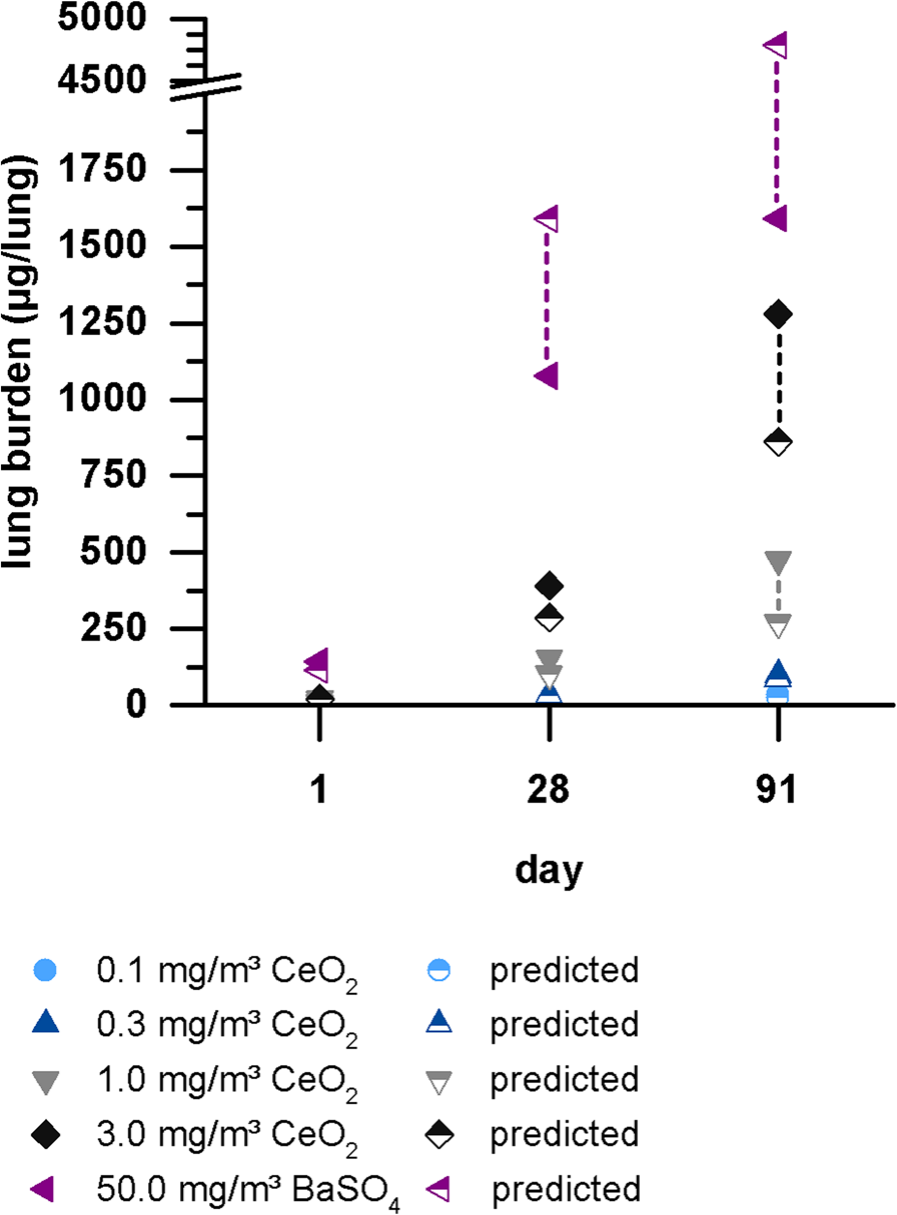
Measured vs. predicted lung burden. Ce and Ba contents were measured in explanted lungs of rats exposed to 0.1, 0.3, 1.0 or 3.0 mg/m^3^ CeO_2_ or 50.0 mg/m^3^ BaSO_4_ nanoparticles for up to 90 days. Predicted values (half colored symbols) were based on deposition fractions calculated via the MPPD model and expected first order elimination with half-times of 70 days (0.1–1.0 mg/m^3^) or 200 days (3.0 and 50.0 mg/m^3^)

Equation (a) was used to calculate the deposited particle mass after one exposure day (6 h), based on the deposition fraction determined with the MPPD model version 2.11 [32]. Equation (b) considered the particle clearance over time for calculating the deposited mass after 28 and 90 days with an exposure rhythm of 6 h/day for 5 days/week. The elimination constant k is based on a standard elimination half-time of 70 days (applied for 0.1, 0.3 and 1.0 mg/m^3^ CeO_2_) or 200 days to mimic lung overload (applied for 3.0 mg/m^3^ CeO_2_ and 50.0 mg/m^3^ BaSO_4_). Development of the predicted lung burden during exposure is illustrated in Fig. 2 and Table 2.

**Fig. 3:**
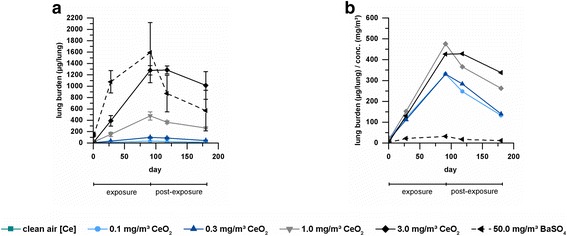
Nanoparticle exposure-related lung burden. Rats were exposed to clean air, 0.1, 0.3, 1.0 or 3.0 mg/m^3^ CeO_2_ or 50.0 mg/m^3^ BaSO_4_ nanoparticles for up to 90 days. Ce and Ba contents were measured in explanted lungs at exposure day one and 28 as well as post-exposure day one (d 91), 28 (d 118) and 90 (d 180). **a** Mean total Ce/Ba content (insoluble + soluble fraction) ± SD, *n* ≤ 5; **b** Mean total Ce/Ba content (insoluble + soluble fraction) normalized to the related initial nanoparticle concentration. Ba contents in the clean air control were at the detection limit and are therefore not shown. The mean insoluble fraction was <5% of the total content

Figure 3 illustrates the lung burden caused by nanoparticle inhalation. It reflects an exposure related increase of Ce or Ba present in the lungs of the exposed animals. Also, the substance deposition was clearly concentration dependent. Particle elimination was visible in all treatment groups after end of exposure. The lower CeO_2_ dose groups (0.1, 0.3 mg/m^3^) as well as the mid and high (1.0, 3.0 mg/m^3^), respectively showed similar development of lung burdens (Fig. 3b). At higher CeO_2_ concentrations higher deposition rates have been detected with reduced elimination especially for 3.0 mg/m^3^ CeO_2_. The barium content decreased quite rapidly compared to cerium and normalized lung burden levels were much lower. Corresponding clearance half-times and exact lung burden values are summarized in Table 3. Half-times were calculated based on Eq. (c).

c $$ Dep(t)= Dep(91)\  x\ {e}^{- kt} $$

**Table 3:** Lung burden and clearance half-times of exposed rats

	Lung burden (μg/lung ± SD)					Clearance t_1/2_ (days)
	d 1	d 28	d 90 + 1rec	d 90 + 28rec	d 90 + 90rec	
Clean air	1.2 ± 1.0	0.6 ± 0.2	1.8 ± 0.8	0.8 ± 0.5	1.3 ± 1.3	-
0.1 mg/m^3^ CeO_2_	2.5 ± 0.8	12.0 ± 2.9	33.1 ± 1.4	24.7 ± 6.1	13.2 ± 3.2	67
0.3 mg/m^3^ CeO_2_	5.4 ± 1.9	33.5 ± 2.8	99.2 ± 10.1	85.1 ± 18.2	41.9 ± 8.8	69
1.0 mg/m^3^ CeO_2_	19.6 ± 5.6	152 ± 37.4	476 ± 74.0	366 ± 24.7	263 ± 15.4	108
3.0 mg/m^3^ CeO_2_	21.0 ± 1.0	391 ± 92.3	1280 ± 82.5	1285 ± 69.9	1013 ± 243	224
50.0 mg/m^3^ BaSO_4_	143 ± 16.3	1078 ± 197	1591 ± 530	871 ± 322	571 ± 358	56

Whereas Dep (91) = retained mass (μg) at post-exposure day 1, Dep (t) = retained mass (μg) after t post-exposure days, t = post-exposure time (days) and k = elimination constant (k = ln(2)/t_1/2_).

Predicted values for Ce deposition during 90 days exposure are quite close to the measured lung retention (Fig. 3). The calculated deposition fraction as well as the expected non-overload or overload conditions after exposure to 0.1 and 0.3 or 3.0 mg/m^3^ nanoparticles, respectively match quite well. Surprisingly, a slightly reduced clearance was detected for 1.0 mg/m^3^. Differences between predicted and measured values increased over time and were greatest for barium. Ubiquitous Ce levels were detected in the clean air control group; the content of Ba was at the detection limit (data not shown). The soluble fraction of Ce or Ba was extremely low (mean values <5%). Hence, the total amount was dominated by the insoluble, particulate fraction, reflecting the low solubility of those nanoparticles.

### Hematology and clinical chemistry

**Fig. 4:**
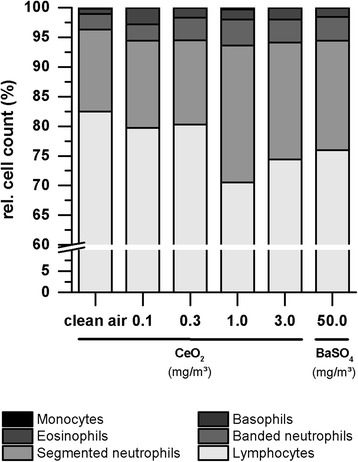
Distribution of blood cells in response to 90 days nanoparticle exposure. Rats were exposed to clean air, 0.1, 0.3, 1.0 or 3.0 mg/m^3^ CeO_2_ or 50.0 mg/m^3^ BaSO_4_ nanoparticles. Blood samples were taken at post-exposure day one. Values are expressed as mean percentage of the total number of cells counted; *n* = 10

Hematological parameters and examinations in clinical chemistry were measured after end of nanoparticle exposure (day 90 + 1rec). Compared to control levels, in the mid (1.0 mg/m^3^) and high (3.0 mg/m^3^) dose group of CeO_2_ the ratio between segmented neutrophils and lymphocytes shifted in favor of increasing neutrophil numbers (Fig. 4). Exposure to BaSO_4_ also caused a slight move of this ratio. The only significant value was measured for neutrophil levels in the CeO_2_ mid dose group. Further blood parameters and biochemical markers measured did not display any significant changes.

### Bronchoalveolar lavages

**Fig. 5:**
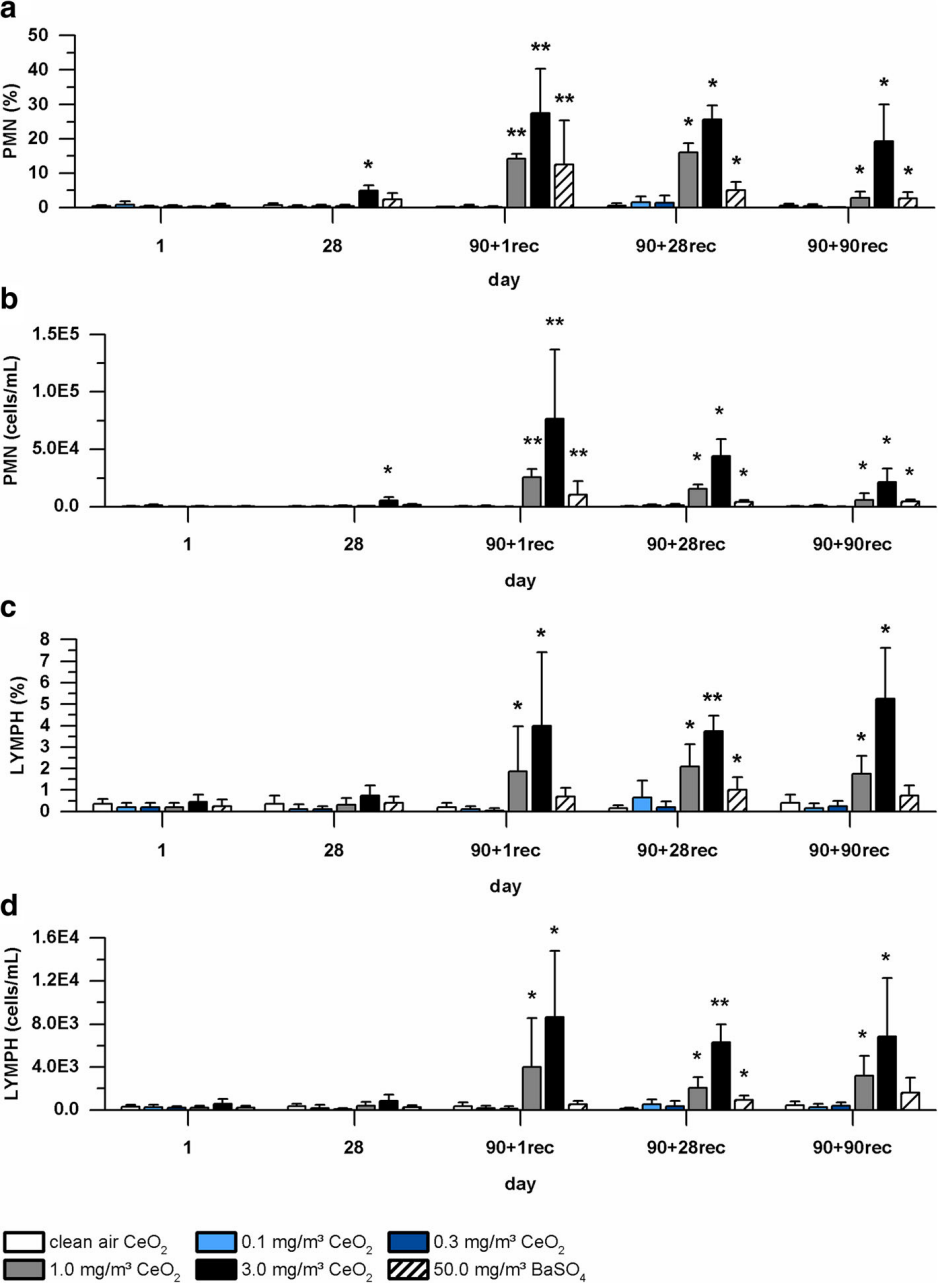
Inflammatory cells measured in BALF. Rats were exposed to clean air, 0.1, 0.3, 1.0, and 3.0 mg/m^3^ CeO_2_ nanoparticles or 50.0 mg/m^3^ BaSO_4_ nanoparticles. **a, b** relative and absolute polymorphonuclear neutrophils (PMN) levels and **c, d** relative and absolute lymphocytes (LYMPH) levels, determined at exposure day one and 28 as well as post-exposure day one, 28 and 90. Values are expressed as percentage of total cell number or absolute value, mean ± SD, * *p* < 0.05, ** *p* < 0.01, *** *p* < 0.001 vs. clean air control, *n* = 5; Kruskal-Wallis-ANOVA with Mann-Whitney U-Test as post-hoc analysis

**Fig. 6:**
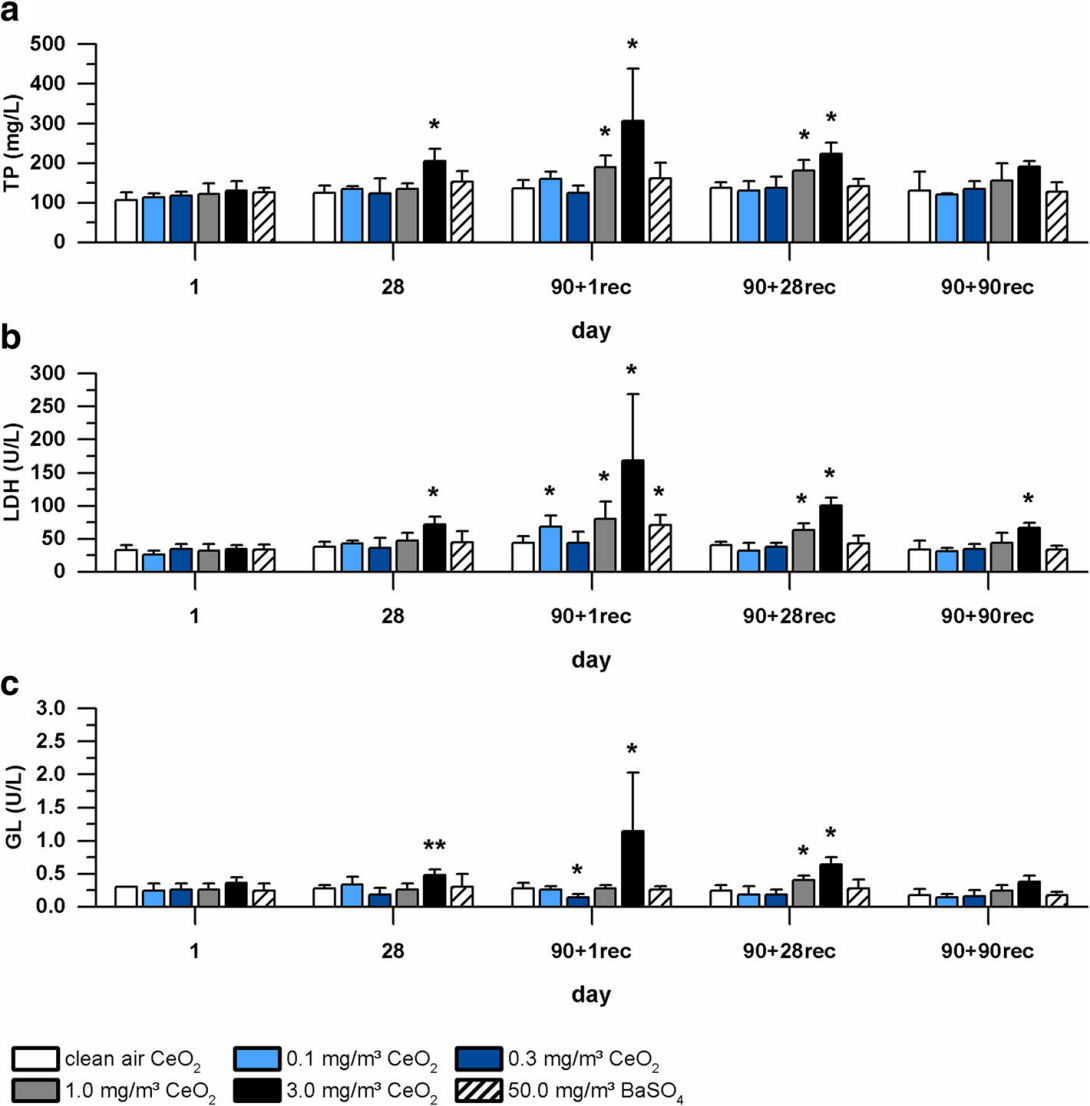
Biochemical parameters measured in BALF. Rats were exposed to 0.1, 0.3, 1.0, and 3.0 mg/m^3^ CeO_2_ nanoparticles and 50.0 mg/m^3^ BaSO_4_ nanoparticles. **a** total protein (TP), **b** lactate dehydrogenase (LDH), and (**c**) ß-glucuronidase (GL) levels were determined at exposure day one and 28 as well as at post-exposure day one, 28 and 90. Values are expressed as mean ± SD, * *p* < 0.05, ** *p* < 0.01 vs. clean air control, *n* ≥ 4; Kruskal-Wallis-ANOVA with Mann-Whitney U-Test as post-hoc analysis

Bronchoalveolar lavage fluid (BALF) was analyzed in all groups after one and 28 exposure days as well as after one, 28 and 90 days post-exposure. A time- and concentration-dependent increase of inflammatory cells, especially neutrophils (PMN) was detected. PMN and lymphocyte (LYMPH) levels increased with ongoing exposure to 1.0 and 3.0 mg/m^3^ CeO_2_ (Fig. 5). No distinct differences between absolute and relative amounts were observed. A slight increase was also detected for total protein (TP), lactate dehydrogenase (LDH) and ß-glucuronidase (GL) levels in the CeO_2_ high dose group. Respective parameters decreased during post-exposure but did not reach control levels until the end of the study (Fig. 6). Described increases of inflammatory cells and biochemical parameters were statistically significant compared to clean air inhalation, especially within the CeO_2_ high dose group. PMN and LYMPH levels were constantly significantly elevated after end of exposure in the mid and high dose group of CeO_2_. Biochemical parameters displayed a more distinct recovery. Elevated levels were significant up to post-exposure day 28 (CeO_2_, mid and high dose group). Both figures clearly illustrate the concentration-dependent impact of cerium oxide nanoparticle exposure as well as time dependency with a distinct peak after 90-day inhalation. Although at lower levels, BaSO_4_ exposure also caused a slight increase of inflammatory cell numbers. However, only PMN levels were significantly higher compared to control levels (Fig. 5a and b). Values clearly decline during post-exposure.

### Histopathology of respiratory organs

Respiratory organs of rats exposed to clean air, 3.0 mg/m^3^ cerium oxide or 50.0 mg/m^3^ barium sulfate were examined histopathologically at all 5 days of sacrifice. Table 4 presents an overview of the most prominent findings with mean grades of severity, separately calculated for all groups and time points considered. Calculations are based on Table 5, which displays the number of incidences with the respective grade of severity for every group and time point. One-time 6 h exposure to CeO_2_ nanoparticles already caused significant accumulations of particle-laden macrophages in the alveolar space and bronchus-associated lymphoid tissue (BALT). The amount of macrophages increased up to the end of post-exposure with translocation to the lung associated lymph nodes (LALN) detected from day 28. Such findings were accompanied by alveolar and interstitial inflammatory cell infiltrations and very slight bronchiolo-alveolar hyperplasia. Free particles (agglomerates) were detected in the alveolar space after end of exposure, mainly in areas of macrophages containing particulate matter. Such accumulations often originate from degrading macrophages. All described pathological conditions remained persistent during 90-day post-exposure. In addition to that, signs of interstitial fibrosis were detected.

**Table 4:**
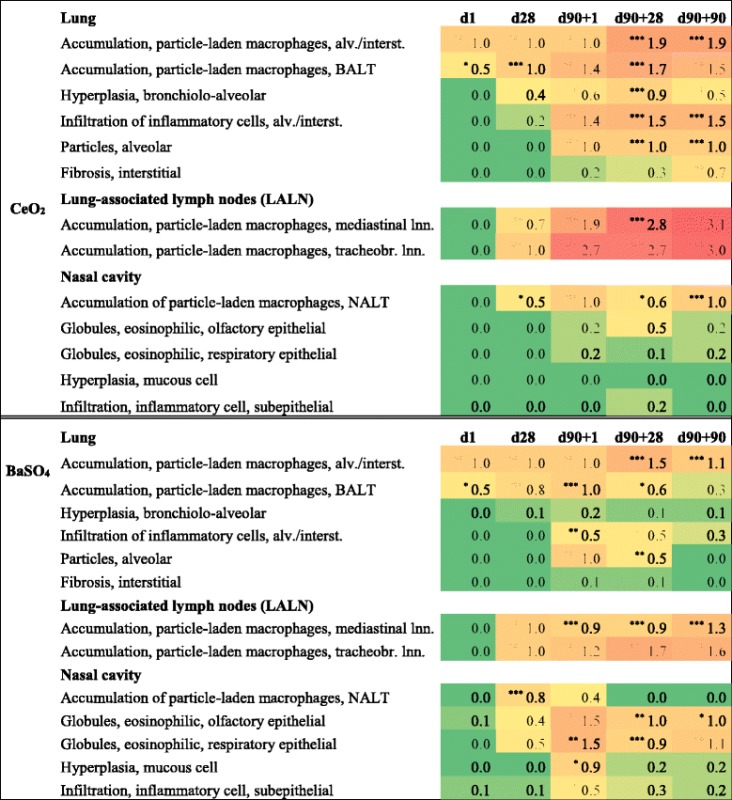
Summary of significant histopathological findings after CeO_2_ and BaSO_4_ exposure

BALT = bronchus-associated lymphoid tissue; NALT = nasal mucosa-associated lymphoid tissue; Values are presented as mean grade of severity: 0 = none, 1 = very slight, 2 = slight, 3 = moderate, 4 = severe (color gradient from green to red indicates increasing severity); n = 9-10; * *p* < 0.05, ** *p* < 0.01, *** *p* < 0.001 vs. clean air control; Group Factor Chi-Squared and Fisher's Exact two sided/Pearson two sided

**Table 5:** Detailed overview of histopathological findings with grade and incidence of effects per animal

Histopathological findings (♀)		Incidence														
Lung		day 1			day 28			day 90 + 1rec			day 90 + 28rec			day 90 + 90rec		
		Ctrl.	CeO_2_	BaSO_4_	Ctrl.	CeO_2_	BaSO_4_	Ctrl.	CeO_2_	BaSO_4_	Ctrl.	CeO_2_	BaSO_4_	Ctrl.	CeO_2_	BaSO_4_
Accumulation, particle-laden macrophages, alveolar/interstitial	examined	10	10	10	10	10	10	10	10	10	10	10	10	10	10	10
	very slight	0	10	10	0	10	10	0	10	10	0	1	5	0	1	9
	slight	0	0	0	0	0	0	0	0	0	0	9	5	0	9	1
Accumulation, particle-laden macrophages, BALT	examined	10	10	10	10	10	10	10	10	10	10	10	10	10	10	10
	very slight	0	5	5	0	10	8	0	6	8	0	5	6	0	6	3
	slight	0	0	0	0	0	0	0	4	1	0	3	0	0	3	0
	moderate	0	0	0	0	0	0	0	0	0	0	2	0	0	1	0
Hyperplasia, bronchiolo-alveolar	examined	10	10	10	10	10	10	10	10	10	10	10	10	10	10	10
	very slight	0	0	0	0	4	1	0	4	2	0	7	1	0	5	1
	slight	0	0	0	0	0	0	0	1	0	0	1	0	0	0	0
Infiltration of inflammatory cells, alveolar/interstitial	examined	10	10	10	10	10	10	10	10	10	10	10	10	10	10	10
	very slight	0	0	0	0	2	0	0	6	5	0	5	5	0	5	3
	slight	0	0	0	0	0	0	0	4	0	0	5	0	0	5	0
Particles, alveolar	examined	10	10	10	10	10	10	10	10	10	10	10	10	10	10	10
	very slight	0	0	0	0	0	0	0	10	10	0	10	5	0	10	0
Giant cells, syncytial, BALT	examined	10	10	10	10	10	10	10	10	10	10	10	10	10	10	10
	present, no grade	0	0	0	0	0	0	0	2	0	0	5	0	0	2	0
Fibrosis, interstitial	examined	10	10	10	10	10	10	10	10	10	10	10	10	10	10	10
	very slight	0	0	0	0	0	0	0	2	1	0	3	1	0	7	0
Lung-associated lymph nodes		day 1			day 28			day 90 + 1rec			day 90 + 28rec			day 90 + 90rec		
		Ctrl.	CeO_2_	BaSO_4_	Ctrl.	CeO_2_	BaSO_4_	Ctrl.	CeO_2_	BaSO_4_	Ctrl.	CeO_2_	BaSO_4_	Ctrl.	CeO_2_	BaSO_4_
Accumulation, particle-laden macrophages, mediastinal lnn.	examined	10	10	10	10	10	10	10	10	10	10	10	10	10	10	10
	very slight	0	0	0	0	7	10	0	2	7	0	0	6	0	0	5
	slight	0	0	0	0	0	0	0	6	1	0	2	1	0	0	4
	moderate	0	0	0	0	0	0	0	1	0	0	8	0	0	9	0
	severe	0	0	0	0	0	0	0	0	0	0	0	0	0	1	0
Accumulation, particle-laden macrophages, tracheobronchial lnn.	examined	10	10	10	10	10	10	10	10	10	10	10	10	10	10	10
	very slight	0	0	0	0	10	10	0	0	8	0	0	4	0	0	4
	slight	0	0	0	0	0	0	0	3	2	0	0	5	0	0	6
	moderate	0	0	0	0	0	0	0	6	0	0	8	1	0	10	0
	severe	0	0	0	0	0	0	0	0	0	0	1	0	0	0	0
Giant cells, syncytial, mediastinal lnn.	examined	10	10	10	10	10	10	10	10	10	10	10	10	10	10	10
	present, no grade	0	0	0	0	0	0	0	4	0	0	7	0	0	10	0
Giant cells, syncytial, tracheobronchial lnn.	examined	10	10	10	10	10	10	10	10	10	10	10	10	10	10	10
	present, no grade	0	0	0	0	0	0	0	8	0	0	8	0	0	10	0
Nasal cavity		day 1			day 28			day 90 + 1rec			day 90 + 28rec			day 90 + 90rec		
		Ctrl.	CeO_2_	BaSO_4_	Ctrl.	CeO_2_	BaSO_4_	Ctrl.	CeO_2_	BaSO_4_	Ctrl.	CeO_2_	BaSO_4_	Ctrl.	CeO_2_	BaSO_4_
Accumulation of particle-laden macrophages, NALT	examined	10	10	10	10	10	10	10	10	10	10	10	10	10	10	10
	very slight	0	0	0	0	5	8	0	10	4	0	6	0	0	10	0
Globules, eosinophilic, olfactory epithelial	examined	10	10	10	10	10	10	10	10	10	10	10	10	10	10	10
	very slight	0	0	1	0	0	4	1	2	3	2	3	8	1	2	4
	slight	0	0	0	0	0	0	0	0	6	0	1	1	0	0	3
Globules, eosinophilic, respiratory epithelial	examined	10	10	10	10	10	10	10	10	10	10	10	10	10	10	10
	very slight	0	0	0	0	0	5	1	2	3	0	1	9	2	2	9
	slight	0	0	0	0	0	0	0	0	6	0	0	0	0	0	1
Hyperplasia, mucous cell	examined	10	10	10	10	10	10	10	10	10	10	10	10	10	10	10
	very slight	0	0	0	0	0	0	0	0	1	0	0	0	0	0	0
	slight	0	0	0	0	0	0	0	0	4	0	0	1	0	0	1
Infiltration, inflammatory cell, subepithelial	examined	10	10	10	10	10	10	10	10	10	10	10	10	10	10	10
	very slight	0	0	1	0	0	1	0	0	5	0	0	1	0	0	2
	slight	0	0	0	0	0	0	0	0	0	0	1	1	0	0	0

*BALT* bronchus-associated lymphoid tissue, *NALT* nasal mucosa-associated lymphoid tissue

**Fig. 7:**
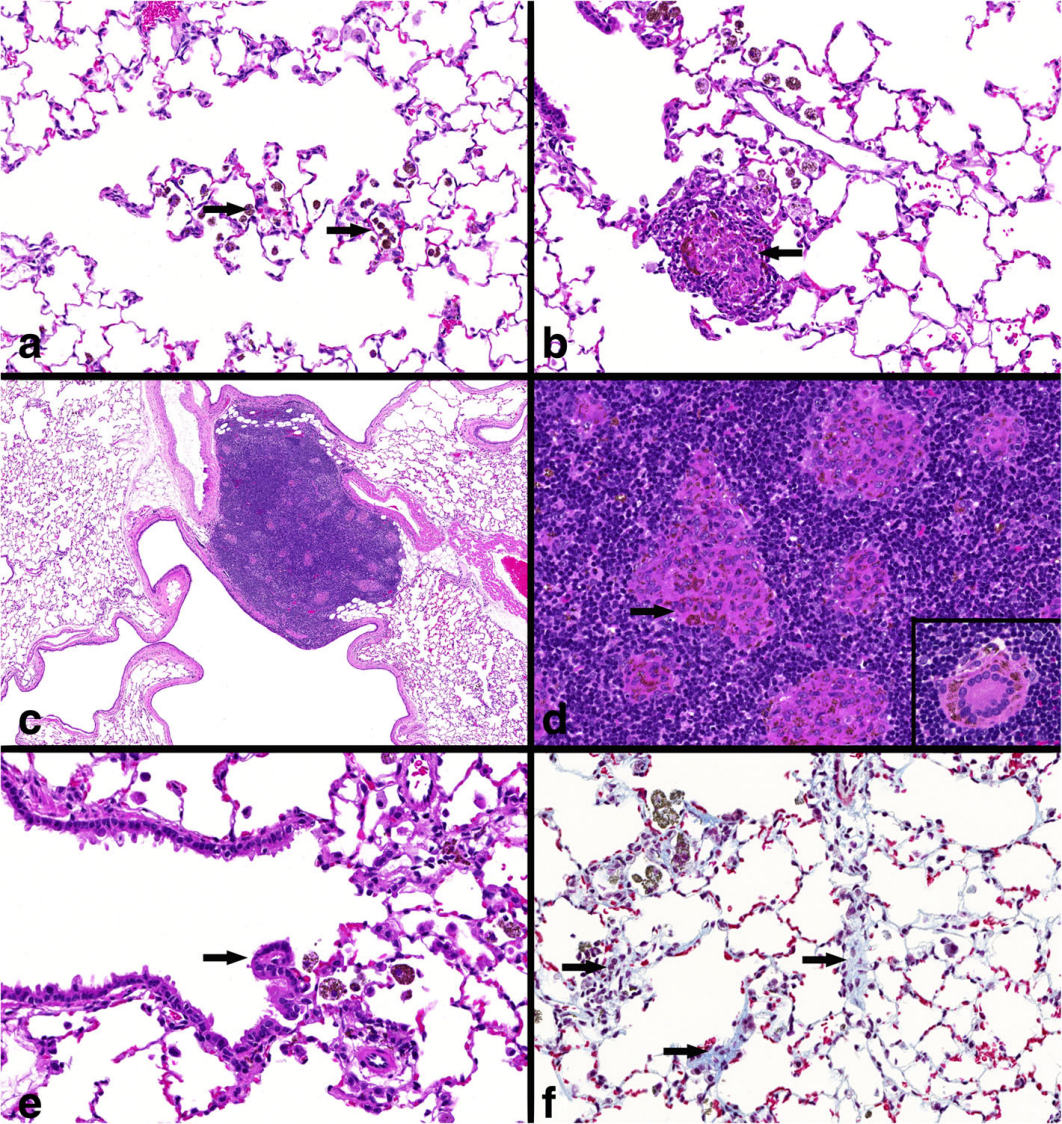
Specific CeO_2_ nanoparticle related histopathological findings. All examples illustrate findings after 3.0 mg/m^3^ CeO_2_ inhalation. **a** lung tissue with particle-laden macrophages (*arrows*), H&E, 33×, **b** inflammatory cell infiltrations with granulomatous inflammation (*arrow*) and alveolar/interstitial particle-laden macrophages, H&E, 37×, **c** bronchus-associated lymphoid tissue (BALT) with foci of particle-laden macrophages, H&E, 5×, **d** foci of particle-laden macrophages in lung-associated lymph nodes (LALN) (*arrow*), H&E, 40×; formation of syncytial giant cells (*insert*), H&E, 50×, **e** focal bronchiolo-alveolar hyperplasia (*arrow*), H&E, 53×, **f** very slight interstitial fibrosis (arrows) and particle-laden macrophages in alveolar tissue, Masson trichome, 40×

Figure 7 displays representative examples of the described particle-laden macrophages, inflammatory cell infiltrations, bronchiolo-alveolar hyperplasia and fibrosis. Alveolar/interstitial foci of macrophages and inflammatory cells were detected. Those infiltrations mainly consisted of lymphocytes and were often located next to bronchioles. Some foci further showed development of a granulomatous inflammation (Fig. 7b). Accumulations of particle-laden macrophages, with syncytial giant cell formation were additionally found in BALT and LALN (Fig. 7c and d). The presence of particle-laden macrophages indicated its migration from the alveolar space to lymphoid tissue for clearance of phagocytosed material. Foci of bronchiolo-alveolar hyperplasia of the bronchiolar type (syn.: alveolar bronchiolization) (Fig. 7e) occurred at very slight (minimal) grade but significant incidence as a result of 90 days nanoparticle exposure. The described pathological findings were accompanied by the development of very slight interstitial fibrosis, significant after 90 days post-exposure (Fig. 7f).

**Fig. 8:**
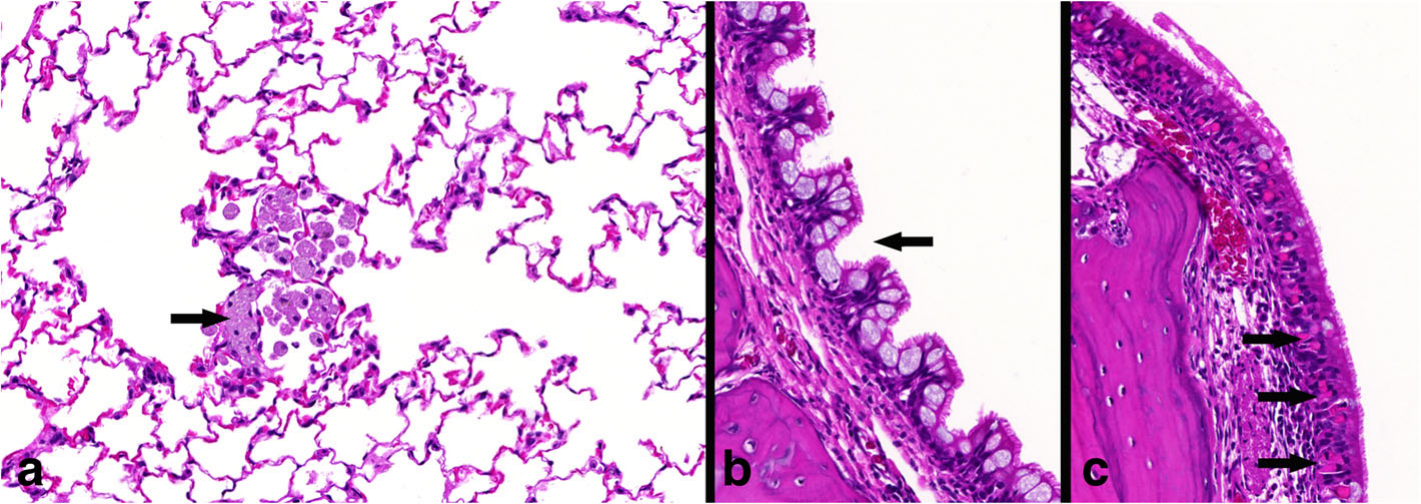
Specific BaSO_4_ nanoparticle related histopathological findings. All examples illustrate findings after 50.0 mg/m^3^ BaSO_4_ inhalation. **a** alveolar tissue with (particle-laden) macrophages (*arrow*), H&E, 37×, **b** mucous cell hyperplasia of the respiratory epithelium in the nasal cavity (arrow), H&E, 40× **c** nasal cavity respiratory epithelia with cytoplasmic eosinophilic globules (*arrows*), H&E, 40×

Effects of 50.0 mg/m^3^ BaSO_4_ exposure were mainly restricted to increasing accumulations of particle-laden macrophages in lung tissue and associated lymph nodes (Table 4 and Fig. 8a). Effects were less severe compared to CeO_2_. Very slight inflammatory cell infiltrations occurred only after 90 days of nanoparticle exposure and did not show any post-exposure persistency. In contrast to CeO_2_, BaSO_4_ nanoparticle inhalation resulted in more distinct pathological changes of the rat’s nasal cavity. Mucous cell hyperplasia and eosinophilic globules in the olfactory and respiratory epithelia were detected from exposure day 28 (Fig. 8b and c). In contrast to the eosinophilic globules, hyperplasia of mucous cells did not remain persistent during recovery.

### Immunohistochemistry

**Fig. 9:**
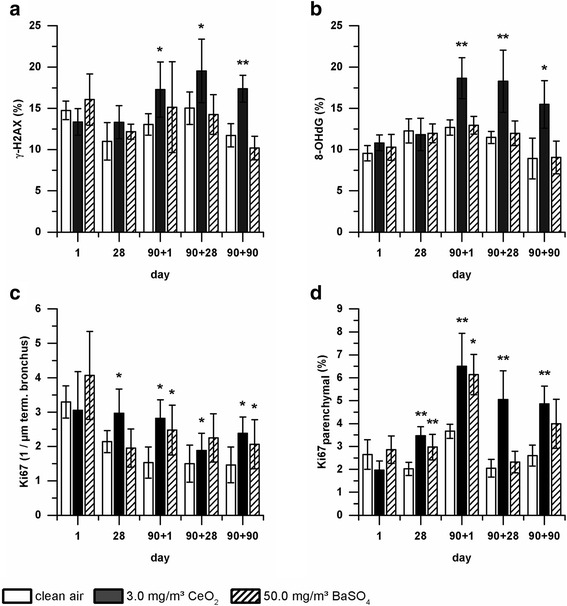
Effects of nanoparticle exposure on immunohistochemistry marker levels in lung tissue. The level of **a** γ-H2AX for genotoxicity, **b** 8-OHdG for oxidative stress, and Ki67 for cell proliferation in **c** terminal bronchi and **d** lung parenchym were determined immunohistochemically in lung tissue of rats exposed to clean air, 3.0 mg/m^3^ CeO_2_ or 50.0 mg/m^3^ BaSO_4_ for one, 28 and 90 days as well as after 28 and 90 post-exposure days. Values are expressed as percentage of positive cells per total cell number or number of positive cells per μm length of terminal bronchus, mean ± SD, * *p* < 0.05, ** *p* < 0.01 vs. clean air control, *n* = 6; Kruskal-Wallis-ANOVA with Mann-Whitney U-Test as post-hoc analysis

To investigate the underlying mechanism of the detected histopathological changes in more detail, immunohistochemical staining of lung tissue for markers related to genotoxicity, proliferation and apoptosis were applied. By this a broad spectrum of potential effects, which have been described in relation to CeO_2_ nanoparticles, was covered. For comparability immunohistochemistry was performed on consecutive slides of lung tissue from the same animals as the described histopathological analysis (clean air, CeO_2_ high dose, and BaSO_4_; all time points). Four markers were selected to determine possible particle-related genotoxicity (Histon γ-H2AX and Hydroxy-2′-deoxyguanosine (8-OHdG)) [33], proliferation (Ki67), and apoptosis (cleaved caspase-3). The latter did not show any changes in nanoparticle exposed animals compared to the control group (data not shown). In contrast, γ-H2AX and 8-OHdG displayed a similar response to CeO_2_ nanoparticle exposure (Fig. 9a and b). Both marker levels were significantly elevated at all measured post-exposure days. Values were consistently about 5% (γ-H2AX) or 6% (8-OHdG) higher than control levels. Ki67 was determined in terminal bronchi and lung parenchym to evaluate proliferative processes in bronchial and alveolar epithelial cells, respectively (Fig. 9c and d). Marker levels were significantly increased after 28 days exposure to 3.0 mg/m^3^ CeO_2_ and remained elevated until the end of the post-exposure period. Interestingly, BaSO_4_ exposure did not reveal elevated marker levels of γ-H2AX and 8-OHdG at any time point investigated but showed significantly enhanced Ki67 levels. However, increased values did not display relevant persistency during post-exposure.

## Discussion

The present lack of data on the (adverse) effects of nanomaterials, especially regarding long-term exposure, requires conduction of appropriate in vivo toxicity studies. For better risk assessment it is necessary to examine realistic concentrations with occupational and environmental relevance. Available in vivo studies on CeO_2_nanoparticles confirm the induction of inflammation after inhalation [3, 6, 17, 22–26]. However, few of them handle concentrations at lower levels (< 3 mg/m^3^) [17, 23, 34]. The main aim of this study therefore was to investigate potential health effects of CeO_2_ nanoparticles after subchronic inhalation to low doses. Because BaSO_4_ is classified as inert dust, low concentrations were not tested further. Instead, effects of BaSO_4_ inhalation at a very high exposure level (50.0 mg/m^3^) were examined to test if there is an upper limit of no effects when administered over longer time spans. Broad investigations after exposure periods of different length, with or without post-exposure recovery yielded information of potential mechanisms of action and provided useful data for potential long-term effects and extrapolation approaches to other nanomaterials.

Consistent aerosol levels were achieved in all dose groups over the whole 90-day exposure with low deviation from the target concentrations. MMAD values for CeO_2_ of 0.7 μm ensured inhalability of aerosols. The dose range selected for CeO_2_ in this study should cover specific conditions of absent inflammation in combination with absent lung overload (0.1 and 0.3 mg/m^3^), inflammation and no overload (1.0 mg/m^3^) as well as inflammation and overload (3.0 mg/m^3^) [17]. Analysis of lung burden suggested that the respective conditions were achieved for the low dose levels and the high CeO_2_ concentration. Exposure to 0.1 and 0.3 mg/m^3^ CeO_2_ resulted in clearance half-times below the expected mean value of 70 days for effective particle clearance [11, 13–15]. Exposure to 3.0 mg/m^3^ CeO_2_ displayed distinct impairment of particle elimination with a half-time > 200 days. This reflects a present overload situation. Keller et al. [17] measured lung burdens of 41 and 520 μg after 28 days exposure to 0.5 and 5.0 mg/m^3^ CeO_2_ NM-212. Furthermore, retention half-times of 40 days were calculated for 0.5 mg/m^3^ CeO_2_ exposure, indicating no impairment of clearance, whereas at higher concentrations lung overload was detected [17]. These values were consistent with our data generated at exposure day 28. Comparable lung burden values have also been detected for other poorly soluble nanomaterials. Bermudez et al. [35] exposed different animal species to 0.5, 2.0 and 10.0 mg/m^3^ nano-TiO_2_ for 90 days and determined retention half-times of 63, 132 and 395 days, respectively in rats. For 1.0 mg/m^3^ CeO_2_ we calculated an increased clearance halftime (t_1/2_ = 108 days). Also, signs of inflammation were present for this dose group. The expected situation of lung inflammation at non-overload was thus not clearly achieved for this concentration. Morrow [12] reflected the overload hypothesis from a volumetric perspective and specified a particulate matter load of 60 μm^3^ per alveolar macrophage as critical value. This corresponds to 1 μL PM/g lung or 1 mg lung burden for particles with 1 g/cm^3^ density. As described by Pauluhn [36] there are great differences between the densities stated for CeO_2_ nanoparticles. He recently evaluated the kinetics of inhaled nano-CeO_2_ NM-212, including data from the long-term study (NANoREG, 81|0661/10|170) and thus the same concentration range and exposure duration as used in our study. He concluded that calculation of volumetric overload for micron-sized particles are applicable also for nano-CeO_2_. His estimations are based on a density of 0.25 g/cm^3^, which would, following the model of Morrow [12], result in lung burden tolerance of 0.25 mg. The lung burden measured in this study after 90 days exposure to 1.0 mg/m^3^ CeO_2_ is above this level and would thus be assumed as overload inducing. However, a reduction of density to 0.25 g/cm^3^ for CeO_2_ as high density material (7.65 g/cm^3^) seems to us a quite great decrease. DeLoid et al. [37] performed comprehensive investigations on density estimation for nanomaterial deposition and determined an effective density for CeO_2_ with different specific surface area in the range of 1.5 to 2.4 g/cm^3^. The higher density would suggest higher volume based lung burden tolerance (1.5–2.4 mg). At this, for us more realistic condition, CeO_2_ would not be related to volumetric lung overload after 90 days exposure, even at a concentration of 3.0 mg/m^3^. Another method to reflect lung overload is based on particle specific surface area. According to Tran et al. [38] the threshold is in the range of 200–300 cm^2^/g lung as measured by PMN recruitment. Application of this method to our particle retention data would reveal surface-related lung burdens of 130 and 360 cm^2^/lung for 1.0 and 3.0 mg/m^3^ CeO_2_, respectively, which is slightly below and slightly above this range. Our half-time measurements compared to the volume- and surface-based overload threshold hypotheses, leave the question in how far the effects, especially of 1.0 g/m^3^ CeO_2_ exposure are caused by lung overload. The relation to particle surface is more consistent with calculated half-times, whereas in contrast to the statement of Pauluhn [36] volume-related overload seems less likely. This suggests that effects are not exclusively overload-related and indicates contribution of particle surface area and chemistry to toxicity. Furthermore, this shows that for accurate interpretation of nanoparticle effects the material’s physico-chemical characteristics should be taken into consideration and the most suitable method for overload calculations should be selected carefully.

In his modelling, Pauluhn [36] determined half-times of 67, 74, 100 and 179 days and a benchmark NOAEL of 0.64 mg/m^3^ (critical parameter: PMN levels in BALF) for 90-day CeO_2_ exposure. Application of our 90-day PMN data revealed a NOAEL_BMDL_ of 0.41 mg/m^3^ (US-EPA benchmark software [39]). Although this NOAEL is slightly lower, our results are all in all consistent with Pauluhn [36] for similar concentrations and study duration.

Less information on NOAELs derived from inhalation studies with nanoparticles exist. This could be attributed to the testing of quite high concentrations. Morimoto et al. [23] stated as the result of a 28 day exposure of rats to 3 mg/m^3^ of nano-ceria (Wako Chemical, Ltd.) a PMN increase persisting over 90 days post-exposure. Pathological features revealed that inflammatory cells, including macrophages and neutrophils, invaded the alveolar space in both studies. Taken together, the CeO_2_ nanoparticles induced a pulmonary inflammation of persisting character. Christensen et al. [40] derived a NOAEL of 0.5 mg/m^3^ for nano-TiO_2_ (based on a multispecies 90-day inhalation study of Bermudez et al. [35]) which is quite close to CeO_2_. In the study of Bermudez et al. [35] pulmonary responses of different species to nano-TiO_2_ (P25; Degussa-Evonik) were compared. Female rats, mice, and hamsters were exposed to aerosol concentrations of 0.5, 2.0, or 10 mg/m^3^ for 90 days. Lesions in the mid-dose group were minimal to mild in severity and consisted primarily of particle-laden macrophage accumulation and aggregation in subpleural regions and in centriacinar zones. These macrophage aggregations were associated with minimal hypertrophy and hyperplasia of type II alveolar epithelial cells. In the high concentration–exposed rats, through 90 days post-exposure, there were progressively more severe epithelial proliferative changes, including metaplastic changes in the centriacinar region (bronchiolization of alveolar epithelium) associated with particle and particle-laden macrophage accumulation. Clearance of particles from the lung was markedly impaired in mice and rats exposed to 10 mg/m^3^ uf-TiO_2_ (not in hamsters). Comparison of the results to the 90-day test with nano-ceria suggests a relatively mild toxicity of both dusts at the 2 and 3 mg/m^3^ concentration, respectively. There are indications for similar no effect levels between different nanoparticles. However, substance specific differences in reactivity are likewise. Differentiation between such findings are important to consider in nanoparticle grouping approaches.

Our measured retained lung loads further match the predicted particle deposition, based on the MPPD model calculations and rat standard breathing parameters. Clearance half-times were chosen based on the expected overload/non-overload conditions for the different dose groups described earlier. Following the results of Keller et al. [17] t_1/2_ = 200 days was selected to reflect lung overload at 3.0 mg/m^3^ CeO_2_. The deposition fraction of about 10% of the initial nanoparticle concentration were quite accurate. Similar values were calculated by Geraets et al. [16]. Respective results verify the predictivity of this calculation method for estimating exposure dose levels prior to animal exposure.

The results of BALF analysis indicated a present inflammatory reaction in the lung after 1.0 and 3.0 mg/m^3^ CeO_2_ exposure. The highest response was measured for neutrophils which was expectable, since neutrophil levels in BALF serve as highly sensitive marker for lung inflammation [41]. The immune reaction is often supported by increased levels of total protein [41], which was observed here as well. Recruited by macrophages for host defense, neutrophils are cells of early inflammatory responses. Although to a much lower level, lymphocyte numbers were also increased. Since most of these cells are responsible for adaptive immune responses occurring in the second instance, the observed development of events is quite consistent. Similar observations for CeO_2_ tested in vivo (28 days exposure) were described earlier [3, 17, 22, 23]. Increased LDH levels in BALF as an indication for cell damage and GL for increased phagocytic activity [41, 42] further supported the detected ongoing elimination of particles by macrophages and associated inflammatory reactions. In addition to the modulations of BALF parameters, increases in the percentage of blood neutrophils were detected. This generally indicates the presence of infections or inflammatory reactions in an organism. Elevated levels thus provide further evidence for the inflammation induced in the lung after 90 days nanoparticle inhalation. Respective values were elevated for CeO_2_ concentrations at which point signs of inflammation were detected in BALF. Increased blood neutrophil numbers have also been measured in other in vivo inhalation studies for the testing of CeO_2_ nanoparticles, including NM-212 [3, 17, 19, 22]. Keller et al. [17] reported increased blood neutrophils after 5 days of exposure to 25 mg/m^3^ CeO_2_ NM-212, but not at lower dose levels. After 4 weeks of inhalation no changes in blood parameters were detected. In our study, blood neutrophil levels were only 5–10% higher after CeO_2_ treatment, compared to the control group. Besides, no other clinical chemistry parameters displayed any abnormalities. Therefore, further blood analysis at a later time point was not performed.

All parameters measured in BALF showed similar trends with post-exposure persistency, especially for the CeO_2_ high dose group. Therefore, broad histopathology examinations were done for this group, clearly confirming the presence of lung inflammation due to nanoparticle inhalation. Immediately after the first exposure interval macrophages with phagocytosed material were detected, over time translocating to lymphoid tissues for particle clearance. This event should not necessarily be rated as an adverse effect, because alveolar macrophages present the normal first line of defense against inhaled foreign material [3, 42]. Granuloma formation and the presence of syncytial giant cells more likely illustrate pathological events and the time-dependent increase of severity by development of a granulomatous inflammation. Respective situations are often caused by oversaturated elimination mechanisms. Our data revealed lung overload at 3.0 mg/m^3^ CeO_2_ nanoparticle exposure. Related impaired macrophage clearance activity could therefore be suggested. Exacerbation of inflammation with ongoing exposure was also seen here by increasing inflammatory cell infiltrations with lymphocytes migrating to the interstitial tissue. Like in BALF analysis this indicates the advanced inflammation reaction. This is also reflected by very slight interstitial fibrosis, usually developing from chronic tissue inflammation. This series of effects illustrates the consequence of particle overload: impaired macrophage activity and particle elimination leads to translocation of particles to the interstitium [15], causing local interstitial effects like inflammatory cell infiltrations or even worse, fibrotic lesions [43, 44]. The impact of nanoparticle inhalation detected here was thus shifting over time from non-adverse to adverse findings. In addition, persistency of effects was measured up to the last day of sacrifice. This suggests an increased risk of long-term effects like tumor development and interstitial fibrosis. Although the grade of interstitial fibrosis was minimal, it results from the ongoing alveolar/interstitial (granulomatous) inflammation induced by CeO_2_ and should be rated as adverse. Especially with respect to long-term exposure such findings may be important for human risk assessment. In most of the in vivo studies mentioned earlier, histopathological investigations were performed, revealing comparable findings after CeO_2_ exposure, especially in terms of particle-laden macrophages and lung inflammation [3, 6, 17, 22, 23, 26]. The described development of inflammatory reactions after inhalation have also been shown for nano-TiO_2_. In the study of Bermudez et al. [35] concentration-dependent increases of inflammatory cells in BALF and histopathological changes comparable to our data were reported. This suggests that the typical behavior of poorly soluble nanomaterial applies for nano-CeO_2_.

More severe histopathological observations as those described here were made rarely. However, this is obvious since such events normally occur at later stages, e.g. in response to prolonged inflammation, and most of the published studies cover short-term setups. The long-term study performed by BASF (Ludwigshafen, Germany) is therefore quite promising regarding the generation of data on this issue. Some information on fibrosis or tumor development are nevertheless available. Ma et al. [45] reported prominent signs of fibrosis after single intratracheal instillation. In contrast, Morimoto et al. [23] did not detect fibrosis or tumor development of lung tissue after single ceria intratracheal instillation or 28-day inhalation with up to 90 days recovery. The absence of effects after inhalation might be due to shorter exposure phases compared to our study. Signs of fibrosis occurred only at very slight grades and late stages of study.

Immunohistochemical analysis of lung tissue was performed to check for additional nanoparticle related molecular events next to inflammation induction. Investigation of the same lung compartments allowed good correlation to our histopathological findings. We found increased levels of genotoxicity and cell proliferation markers in response to 3.0 mg/m^3^ CeO_2_ nanoparticle exposure. Although significantly, values were just slightly exceeding the control level and should be interpreted with reservation. Interestingly, similar to inflammatory events, effects remained stable and did not recover up to the end of the 90 day post-exposure period. It is known that the three events inflammation, genotoxicity and cell proliferation are crucial in carcinogenesis. Particles are suggested to affect the underlying molecular mechanisms, as it has early been reviewed by Oberdörster [15]. Activated inflammatory cells, including neutrophils and macrophages release reactive oxygen species (ROS) and growth factors during particle elimination. This increases the risk of occurring genotoxic and proliferating processes in target cells and promotes tumor development. It is evident that this is even more critical in situations of persistent inflammation due to lung overload. Respective mechanisms might also cause lung fibrosis [15] which has been observed in this study at very slight levels. Higher grades of interstitial fibrosis after extended nanoparticle exposure or even longer post-exposure periods could be suggested. However, this must be verified in continuing studies. Potential genotoxic effects have been investigated for many particles, including poorly soluble, and especially those with pro-carcinogenic activity [46]. Significant increases in 8-OHdG together with changes of Ki67 levels have been described for quartz particles [47]. Correlations between the genotoxicity marker and tumor development after exposure to diesel exhaust particles has been detected by Ichinose et al. [48]. Most of the published in vivo studies on CeO_2_ did not report investigations on genotoxicity. Larsen et al. [25] examined short-term exposure to a group of metal oxide nanoparticles including CeO_2_ and detected signs of DNA damage in lung tissue only after TiO_2_ inhalation. Keller et al. [17] reported absent systemic genotoxicity at early stages of exposure to CeO_2_ NM-212 (five and 28 days), but high concentrations of up to 25 mg/m^3^. In the corresponding long-term study, genotoxicity in blood cells was investigated by three different assays after 3 and 6 month CeO_2_ or BaSO_4_ exposure periods without any positive findings [18]. Authors concede that this effect could indeed be due to an absent genotoxic potential of the particles, but also, particle translocation too low to cause any measurable extra-pulmonary effects could be the reason. Although we did not test systemic genotoxicity in our study, the absent effects reported by Cordelli et al. [18] indicate a restriction of effects to pulmonary organs. In contrast, systemic genotoxic effects were reported after single and repeated oral administration of CeO_2_ [49, 50]. Considering that respective findings were present only at high concentrations of >300 mg/kg BW, a potential for the induction of DNA damage via secondary rather than primary genotoxic mechanisms could be assumed for CeO_2_ nanoparticles. This is further supported by in vitro testing of CeO_2_ NM-212 revealing genotoxic effects at non-cytotoxic levels in different cell lines [51].

The induction of programmed cell death is an opponent of increased cell proliferation and tumor development. Absent changes of cleaved caspase-3 levels for the duration of the study further support the potential effect relationships. Elevated cleaved caspase-3 levels have been reported after CeO_2_ nanoparticle instillation [30]. Since a much higher concentration was applied compared to our study, the induction of apoptosis was likely caused by this single high substance exposure event, while continuous contact of lung tissue to lower nanoparticle concentrations do not affect this pathway of programmed cell death. Our current findings were thus quite consistent: enhanced risk of lung fibrosis or tumor development could be suggested, considering the distinct, persistent lung inflammation mediated by neutrophils and macrophages with evidence of increased cell proliferation in terminal bronchi and lung parenchyma as well as increased DNA damage of lung epithelial cells at a CeO_2_ nanoparticle concentration inducing high levels of lung burden with impaired clearance.

The interpretation of our findings match the hypothesis of particle-related carcinogenesis [15]. Respective inflammation based mechanisms and the role of primary genotoxicity are still intensively discussed [46, 52]. We must exclude primary mechanisms for the high CeO_2_ dose tested here, because we demonstrated inflammation and particle overload. To evaluate additional carcinogenicity mechanisms, which are based on direct interaction of nanoparticles with cellular compartments, concentration levels ≤0.3 mg/m^3^ should be further evaluated with respect to ROS formation, genotoxicity, increased cell proliferation and apoptosis.

BaSO_4_ exposure revealed some significant findings in our study although this substance is assumed to be chemically inert and non-toxic. It has to be taken into consideration that a very high concentration was tested here, which is of less relevance for mimicking certain exposure scenarios. To understand possible mechanisms of action of BaSO_4_ nanoparticles, the generated data of high dose exposure is nevertheless quite useful. BaSO_4_ was cleared rapidly and therefore differs from other poorly soluble nanomaterials. Lung burden values of CeO_2_ and BaSO_4_ were similar after 90 days exposure. In contrast, BaSO_4_ clearance half-time was much lower and exposure-related effects were less severe even though the concentration was up to 500-times higher compared to CeO_2_. Similar findings have been stated by others [6, 20]. Konduru et al. [20] attributed substance-specific characteristics and fast clearance to the differences in toxicity. Characteristics like dissolution, shape, and agglomeration state are known to influence the toxic potential of nanomaterials. Like it is known for comparable metals, a considerable part of inhaled BaSO_4_ translocates to bones [20]. It remains unclear in how far ionic barium, and therefore dissolution contributes to rapid clearance and translocation. Although slow dissolution has been suggested, Konduru et al. [20] consider structural changes and related switches in surface charge as reason for unexpected increases of dissolution rates and resulting rapid clearance. The high MMAD of 2.95 μm determined for BaSO_4_ (compared to CeO_2_: approx. 0.7 μm) indicated agglomeration. Consequently, BaSO_4_ deposited in the upper respiratory tract, which was also reflected by the low predicted alveolar deposition fraction. Major histopathological effects were thus found in the nasal cavity while inflammatory reactions in the alveolar compartments were marginal. The agglomeration potential might explain substance-related differences in toxicity between both nanoparticles but to a certain amount this also depends on the high amount of particles administered.

Differences in clearance rates were also detected in comparison to TiO_2_, another poorly soluble dust [38]. The more effective elimination of BaSO_4_ was explained by a lower specific surface area. Although the tested particles were micron-sized this indicates that BaSO_4_ behaves differently compared to TiO_2_ and CeO_2_ when entered the respiratory tract. As it has been discussed above, clearance rates of nano-TiO_2_ and nano-CeO_2_ were comparable [35].

Based on short-term exposure to nanoparticles a NOAEC of 50.0 mg/m^3^ for BaSO_4_ NM-220 was stated [6]. After 90-day inhalation of the same concentration we found several effects supporting refute of this limit value. Although there was no distinct inflammatory reaction with granulomatous characteristics like it was present after CeO_2_ exposure, we found elevated levels of neutrophils in BALF. Post-exposure histopathology examinations revealed remaining persistency of slight cell accumulations especially in mediastinal and tracheobronchial lymph nodes. Even more distinct was the presence of eosinophilic globules within respiratory and olfactory epithelial cells of the nasal cavity and very slight mucous cell hyperplasia. No signs of genotoxicity or apoptosis were detected. Slightly increased Ki67 positive cell counts measured after 90-day exposure indicated proliferative effects. However, return of values to control levels during post-exposure suggested low relevance of the respective findings. Absence of genotoxic effects, in this case systemic, were also stated by Cordelli et al. [18] using the same BaSO_4_ nanoparticles and the same concentration. The adverse effects caused by BaSO_4_ exposure could be a consequence of high nanoparticle levels and agglomeration especially in the upper respiratory tract. However, the differences in clearance and toxicity between BaSO_4_ and CeO_2_ suggest contribution of substance inherent characteristics (e.g. surface conditions). Those findings further show that BaSO_4_ differs from other poorly soluble particles. This should be considered regarding grouping approaches and risk assessment.

## Conclusion

CeO_2_ nanoparticles reach the alveolar space and induce persistent inflammatory reactions after inhalation with a NOAEL below 1.0 mg/m^3^. There are indications for overload-related inflammatory effects. However, particle specific toxicity, likely related to surface area is suggested and has to be proven in future studies. Inflammatory effects of BaSO_4_ are mainly restricted to the nasal cavity, less severe and persistent compared to CeO_2_ and most likely related to the high dose level. The rapid clearance of BaSO_4_ discussed in the literature has been confirmed during our experiments. The present study revealed important information on the pulmonary toxicity of CeO_2_ and BaSO_4_ nanoparticles. It provides useful data for nanomaterial risk assessment and possible approaches on grouping. Further mechanistic evaluations are required especially regarding potential genotoxic effects and the role of oxidative stress in CeO_2_ nanoparticle reactivity.

## Methods

### Nanoparticles

Cerium oxide NM-212 and barium sulfate NM-220 were provided by the Fraunhofer Institute for Molecular Biology and Applied Ecology (Fh-IME, Schmallenberg, Germany). Both nanoparticles belong to the European Commission Joint Research Center (JRC) Nanomaterial Repository (Ispra, Italy).

***CeO***_***2***_ ***NM-212:*** primary particle size 28.4 nm, mean BET surface area 27.2 m^2^/g, water solubility <1 μg/L, purity >99.5% (Information provided by Sigh et al. [53] and Fh-IME Schmallenberg).

***BaSO***_***4***_ ***NM-220:*** primary particle size 37.5 nm, mean BET surface area 41.4 m^2^/g, water solubility 0.6 × 10^−3^ w-% Ba^++^, purity >93.8% (Information provided by Wohlleben et al. [54] and Fh-IME Schmallenberg).

### Animals

Female Wistar rats [Crl:WI (Han)] were purchased from Charles River (Sulzfeld, Germany) and kept in groups of two animals in Makrolon polycarbonate cages Type IV. Subsequent to 1 week of acclimatization rats were habituated to nose-only tubes for 3 weeks, randomized and finally exposed to clean air or test substances with a start age of 10 weeks. Temperature of animal rooms was set at 20–24 °C with 40–70% relative humidity and a light/dark cycle of 12 h. Laboratory diet (“V1534”, sniff Spezialdiäten GmbH, Soest, Germany) and water was supplied ad libitum. All experiments were conducted and approved according to the German Animal Welfare Act by the local authority at the LAVES Niedersachsen, Hannover, Germany, No. 33.12–42,502–04-14/1564.

### Exposure atmosphere

Aerosols were generated by dry powder dispersion using a high-pressurized, high velocity pressurized air dispersion nozzle developed at our Institute [55]. Briefly, the test material was located in reservoirs on a rotating disc and sucked into the air flow system. Different nanoparticle concentrations were achieved by adjusting the feed rate via rotational speed regulation. Control group animals were provided with clean air. Generated aerosols were introduced into a nose-only inhalation system. Aerosol concentrations were continuously recorded by a light scattering aerosol photometer (Fraunhofer ITEM, Hannover, Germany) and compared to additional filter sample analysis. The nanoparticle’s MMAD was determined independently for each group by gravimetric analysis (Marple 298 Personal Cascade Impactor, Thermo Fisher Scientific). Exposure tube positions were changed daily to minimize differences due to geometry.

### Study design

The in vivo 90-day inhalation toxicity study was conducted according to OECD TG 413 [56]. CeO_2_ NM-212 was administered in concentrations of 0.1, 0.3, 1.0 and 3.0 mg/m^3^, BaSO_4_ NM-220 in one high concentration of 50.0 mg/m^3^. A total of 576 rats were exposed to clean air or the test substances for up to 90 days in a 6 h/day, 5 days/week rhythm. Clinical examinations were performed after one and 28 days of exposure as well as after one, 28, and 90 days post-exposure period.

### Clinical signs, food consumption and body weights

The health condition of animals was checked daily. Broad inspection for clinical abnormalities outside of the cage were done once a week. On exposure days clinical observations were done before, after and if necessary during exposure. Food and water consumption was recorded weekly for a representative subgroup of ten animals from each dose group. Body weights of all animals were checked once a week.

### Clinical examinations

#### Hematology and clinical chemistry

Ten animals of each dose group were used for hematological and clinico-chemical examinations at post-exposure day one. Blood was taken by puncture of the retrobulbar venous plexus under slight isoflurane anesthesia. Full blood analysis and clinical chemistry parameters were recorded according to OECD TG 413 requirements [56].

#### Lung burden

In order to determine the lung retention of CeO_2_ and BaSO_4_ five animals of all dose groups were examined at all days of sacrifices. Explanted lungs of exposed animals were separated and the right lobes were used for analysis of lung burden. The isotopes ^140^Ce/^142^Ce and ^135^Ba/^137^Ba in organ samples were quantified via inductively coupled plasma mass spectrometry (ICP-MS) using a quadrupole ICP-MS system (X-Serie II, Thermo Fisher Scientific). Sample preparation included lyophilisation of shredded tissue for at least 6 h (0.37 mbar). Organ weights were recorded prior and subsequently to freeze-drying. For removal of organic material samples were further processed by plasma ashing (cool plasma conditions, 400 W, 1 mbar O_2_, 24 h) and subsequent microwave digestion (H_2_SO_4_, 96%, supra quality, max. 500 W).

#### Bronchoalveolar lavage analysis

Bronchoalveolar lavages (BAL) of rat lungs were performed in five animals of each dose group at all five time points. The method is based on Henderson et al. [57] with minor modifications. Lungs were lavaged twice using 4 mL 0.9% NaCl. The following parameters were determined from collected lavage fluids: total cell count, differential cell count (macrophages, neutrophils, eosinophils and lymphocytes), biochemical mediators (lactic dehydrogenase, ß-glucuronidase and total protein), as well as cytokine levels. Total cell counts were measured using a counting chamber (Fuchs-Rosenthal). Differential cell counts were prepared by centrifugation of BAL fluid on cytoslides and subsequent Giemsa staining. Biochemical indicators were determined in the supernatant of centrifuged BAL fluid according to routine clinical chemistry protocols.

#### Histopathology

All organs and tissues were preserved and wet weights were recorded according to OECD TG 413 [56]. Animals were killed by carbon dioxide overdose and subsequent exsanguination. Histopathological examinations of respiratory organs were performed at all implemented days of sacrifice in ten animals of the clean air control, 3.0 mg/m^3^ CeO_2_ and BaSO_4_ group respectively. Left lung lobes including bronchi as well as mediastinal and tracheobronchial lung-associated lymph nodes, trachea, pharynx and nasal cavities including nasal mucosa-associated lymphoid tissue were investigated. All respiratory tract organs were fixed in formalin (10%) for 24 h and trimmed according to Ruehl-Fehlert et al. [58], Kittel et al. [59] and Morawietz et al. [60]. The left lung lobe was inflated with formalin (10%) at 20 cm water pressure prior to formalin fixation. After trimming tissues were embedded in paraffin, sectioned, and hematoxylin and eosin (HE) stained for analysis Additionally, Masson trichrome staining of the lung was done for detection of connective tissue production.

#### Immunohistochemistry

In addition to obligatory investigations according to OECD TG 413 [56] immunohistochemical stainings of lung tissue sections from six animals of the clean air control, 3.0 mg/m^3^ CeO_2_ and BaSO_4_ dose group were performed after one and 28 exposure days, as well as after one, 28 and 90 post-exposure days. Samples were prepared as described for histopathology. Histopathological and immunohistochemical analysis was done in consecutive lung tissue sections. Antibodies directed against γ-H2AX and 8-OHdG were used as markers for genotoxicity, caspase-3 for apoptosis, and Ki67 was used to determine lung cell proliferation as described by Rittinghausen et al. [33].

### Statistics/data evaluation

Animal related “in live” data as well as hematological, clinical chemistry, and histopathological findings were recorded using the PROVANTIS 8431 software. Evaluation of body weights, food and water consumption as well as hematology data was done within PROVANTIS 8431, applying ANOVA with Dunnett post-hoc comparison. Bronchoalveolar lavage parameters and immunohistochemistry marker levels were statistically evaluated using Kruskal-Wallis-ANOVA with Mann-Whitney U-Test as post-hoc analysis. Histopathological findings were analyzed by a two-tailed Fisher test in the PROVANTIS 8431 software system.
